# Evolutionary Trends of the Pharyngeal Dentition in Cypriniformes (Actinopterygii: Ostariophysi)

**DOI:** 10.1371/journal.pone.0011293

**Published:** 2010-06-24

**Authors:** Emmanuel Pasco-Viel, Cyril Charles, Pascale Chevret, Marie Semon, Paul Tafforeau, Laurent Viriot, Vincent Laudet

**Affiliations:** 1 Evo-devo of Vertebrate Dentition, Institut de Génomique Fonctionnelle de Lyon, Université de Lyon, CNRS, INRA, Ecole Normale Supérieure de Lyon, Lyon, France; 2 Molecular Zoology, Institut de Génomique Fonctionnelle de Lyon, Université de Lyon, CNRS, INRA, Ecole Normale Supérieure de Lyon, Lyon, France; 3 iPHEP, CNRS UMR 6046, Université de Poitiers, Poitiers, France; 4 European Synchrotron Radiation Facility, Grenoble, France; Centre for Genomic Regulation (CRG), Universitat Pompeu Fabra, Spain

## Abstract

**Background:**

The fish order Cypriniformes is one of the most diverse ray-finned fish groups in the world with more than 3000 recognized species. Cypriniformes are characterized by a striking distribution of their dentition: namely the absence of oral teeth and presence of pharyngeal teeth on the last gill arch (fifth ceratobranchial). Despite this limited localisation, the diversity of tooth patterns in Cypriniformes is astonishing. Here we provide a further description of this diversity using X-ray microtomography and we map the resulting dental characters on a phylogenetic tree to explore evolutionary trends.

**Results:**

We performed a pilot survey of dental formulae and individual tooth shapes in 34 adult species of Cypriniformes by X-ray microtomography (using either conventional X-ray machine, or synchrotron microtomography when necessary) or by dissecting. By mapping morphological results in a phylogenetic tree, it emerges that the two super-families Cobitoidea and Cyprinoidea have followed two distinct evolutionary pathways. Furthermore, our analysis supports the hypothesis of a three-row dentition as ancestral for Cyprinoidea and a general trend in tooth row reduction in most derived lineages. Yet, this general scheme must be considered with caution as several events of tooth row gain and loss have occurred during evolutionary history of Cyprinoidea.

**Significance:**

Dentition diversity in Cypriniformes constitutes an excellent model to study the evolution of complex morphological structures. This morphological survey clearly advocates for extending the use of X-ray microtomography to study tooth morphology in Cypriniformes. Yet, our survey also underlines that improved knowledge of Cypriniformes life traits, such as feeding habits, is required as current knowledge is not sufficient to conclude on the link between diet and dental morphology.

## Introduction

Many biological structures such as somites, segments or limbs, exhibit repeated patterns and these structures are often variable between related species. Within these serially homologous structures, teeth were relatively neglected by Evo/Devo studies despite their promise for describing and understanding both the diversity and evolution of complex adaptive structures. Teeth can be used for integrated studies and results have begun to accumulate on the genomic and/or developmental basis of their wide diversity [1], [2]. Up to now, the evolution and development of teeth have been more intensively investigated in the mouse model in which mechanisms controlling tooth crown shape, tooth identity, dental row segmentation, or occurrence of toothless areas are under intense investigation [1]–[3]. However, teeth of Actinopterygians are becoming more and more intensively studied [4], [5]. When compared to Mammals, Actinopterygian fish display two dental traits that are reminiscent of the basal condition of Vertebrates: (*i*) their teeth are widely distributed within the oral and pharyngeal cavities, and (*ii*) their teeth are constantly replaced throughout the duration of the animal's life (polyphyodonty) [6], [7]. Interestingly, Actinopterygian fish display a great diversity in tooth number and location [4], [8]: they display rows of hundreds of teeth which can be located on the lower and upper jaws, the floor of the mouth (basihyal, basibranchials), the roof of the mouth (e.g. vomer, palatine), the upper and lower pharyngeal regions. In addition to this diversity, Actinopterygian fish include several well-studied experimental models that allow for the study of tooth development from a mechanistic perspective [9]–[11]. This is for example the case of the zebrafish, *Danio rerio*, a member of the order Cypriniformes (Actinopterygii, Osteichthyes) that comprises an excellent model for developmental biology studies [4], [12], [13]. But this is also the case of the Japanese medaka (*Oryzias latipes*), or the Mexican cave fish (*Astyanax mexicanus*), and the Cichlids that harbour an extensive diversity of tooth shapes [10], [11], [14]–[16].

The order Cypriniformes (Actinopterygii, Osteichthyes) encompasses more than 3000 recognized species completely restricted to freshwater and separated in two super-families: Cyprinoidea (including 2 families–Psilorhynchidae and Cyprinidae) and Cobitoidea (including 4 families–Catostomidae, Gyrinocheilidae, Cobitidae, Balitoridae) [8], [17], [18]. Therefore, Cypriniformes constitute an excellent example of a highly diverse clade with huge species diversity. Despite this diversity, all known Cypriniformes present pharyngeal dentition attached to the fifth ceratobranchial [4] and do not develop oral teeth. The nature of the differences in inductive signals that explain the loss of oral teeth in Cypriniformes is currently a question of intense investigation [15], [19]. As no oral teeth have ever been reported in any Cypriniformes [8], [20], it has been proposed that there might exist a developmental constraint impeding the ability to form teeth in the oral cavity in Cypriniformes, as a consequence of a complex series of genetic modifications [4], [15], [19]. Besides its peculiar location, the dentition of Cypriniformes provides a very nice Evo/Devo model for two main reasons: (i) Cypriniformes include the zebrafish which is the model species for Osteichthyans and the most widely used model for Teleost fish; (ii) Cypriniformes exhibit a striking diversity in feeding habits, habitat and size, which is well adapted for investigating the relations between life history traits and the dentition [17], [21].

Several studies aiming at characterizing the developmental pathways that control tooth development in the zebrafish have been recently carried out [15], [16], [19]. The zebrafish dentition is composed of 11 spoon-shaped teeth organized in three distinct rows: the dorsal row with 2 teeth, the mediodorsal row with 4 teeth and the ventral row with 5 teeth. This typical organization can be summarized as follows in the dental formula: 2,4,5-5,4,2 (tooth number is the same on the left and right ceratobranchials, which is not the case for all Cypriniformes as discussed below). This dental formula doesn't take into account replacement teeth that are not bound to the pharyngeal bones when they develop, but only later when they become functional [12], [13].

Many zoological data show that both tooth number and tooth shape are highly diversified within Cypriniformes [4], [8], [17], [18]. Thus, studying the range of possible variations may provide useful indications of the underlying genetic mechanisms controlling tooth shape and distribution [22]. The diversity in tooth number and shape appears especially high in the Cyprinoidea [4], [8], [17] that encompasses well-known species such as the carp (*Cyprinus carpio*) or the goldfish (*Carassius auratus*) in addition to the zebrafish. But this variation is not restricted to the Cyprinoidea as most Cypriniformes exhibit variations in tooth number and/or tooth shape. For example, Cobitidae display about 10 to 20 teeth per row whereas some Catostomidae display about 100 teeth per row [8]. Furthermore, cases of asymmetric dentition patterns between left and right fifth ceratobranchials have been described [4]. Thus, Cypriniformes offer the combination of a well-known and workable model presenting a huge variability of dentition patterns in species that can often be bred in captivity [17], two features that make them attractive models for Evo/Devo studies. In addition, because a large number of economically relevant freshwater fish are Cypriniformes, their phylogeny has been well-studied, compared to other teleost fishes, using whole mitochondrial genome and nuclear markers [23]–[32]. The phylogeny of Cypriniformes now harbors well resolved branches that are supported by high confidence values. The order is split into two main lineages-the Cobitoidea and the Cyprinoidea-each of which is organized into several well-defined families and sub-families whose relationships are partially understood.

If large amounts of data are available on tooth shape and tooth row organization in Cypriniformes [33]–[39], no detailed large scale study describing the extent of the variation at the level of the whole order has been published. From the available dataset, several key questions have begun to be addressed. The number of tooth rows in the ancestral Cyprinoidea dentition has been subject to intense debate: several authors have proposed an ancestral three-row dentition and a persistent trend in reduction of tooth number through time [4], [33], [40], [41]. In addition, Cypriniformes species exhibit strong variations in size. The sub-family Rasborinae displays several independent cases of evolution towards extreme reduction of size, thus providing the opportunity to link body size with the complexity of the tooth pattern [27], [42]–[44].

Here, we characterized the diversity of tooth number and shape within Cypriniformes by imaging pharyngeal teeth in 34 species spread over the major taxonomic units of the order. Previous studies of pharyngeal teeth have been carried out by clearing and staining specimens [45] as well as by conventional dissection to extract the pharyngeal bones [33]. This latter technique can be done by researchers that have gathered some experience. It is also difficult to perform on small species like minute Rasborinae. Moreover, it is destructive, which constitutes a problem for studying rare species, even though we did not include a rare specimen in this present study. In order to overcome these problems, we performed X-ray microtomography to visualize pharyngeal dentitions for 31 Cypriniformes species. Conventional and synchrotron X-ray microtomography are non-invasive techniques that allow performing high quality 3D virtual dissection and rendering of dentitions on a large size range without damaging the specimens [46]. These two techniques are both based on X-ray radiographies taken during the rotation of the samples. Conventional machines can already provide high quality data for many kinds of samples, but in our case, synchrotron imaging was required to image the smallest samples or to reach a higher quality, using some propagation phase contrast, when the conventional data were not good enough (See materials and methods for acquisition and processing details). We also carried out dissection for 3 Cypriniformes species and we used available literature for characterizing pharyngeal dentition of 15 other Cypriniformes species in order to obtain a better view of dentition organization within the whole order. As previous studies have shown that intraspecific polymorphism of tooth number and tooth shape is very low [4], [47], we scanned only one specimen for each species. We reconstructed 3D shapes of tooth rows and we generated a plate representing the pharyngeal dentition in three standards views (occlusal, dorsal, ventral).

## Results

**Figure 1:**
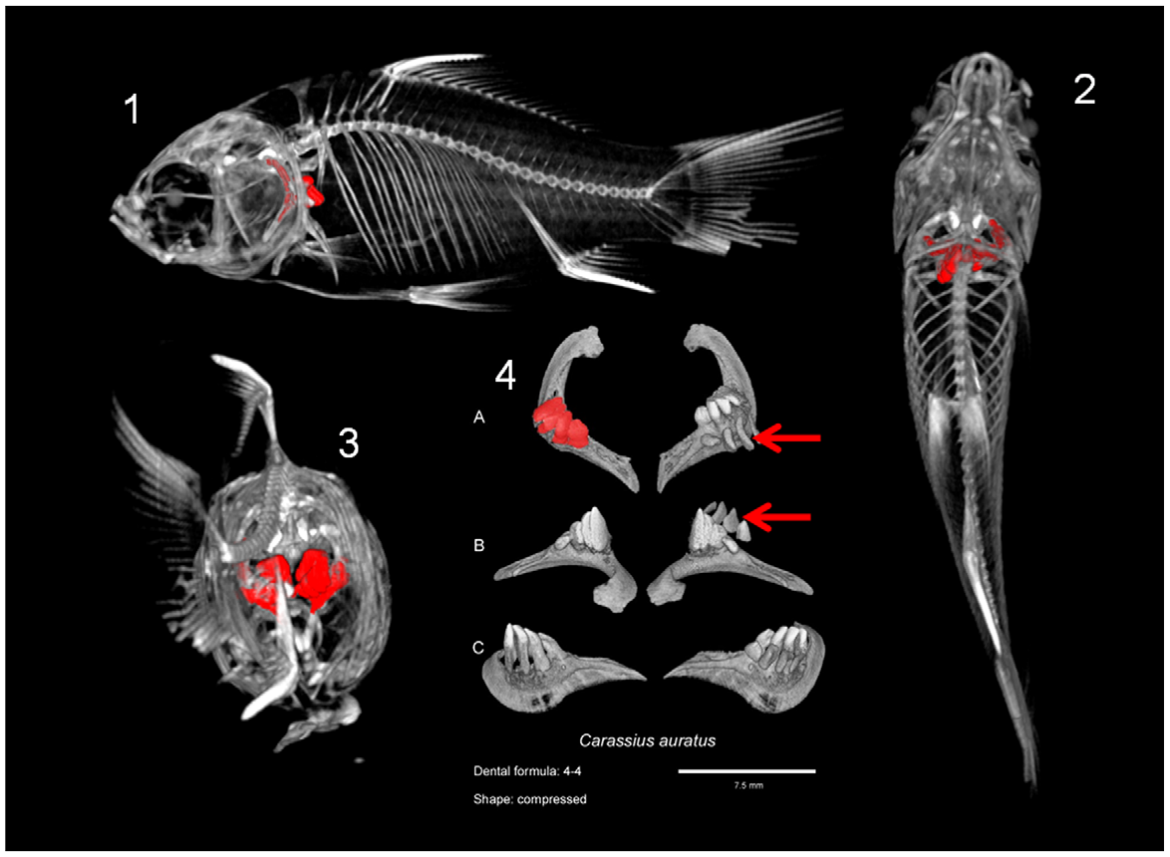
Localization of the fifth ceratobranchial in the goldfish, *Carassius auratus* (Cyprinidae) and dental plate for this species. 1, 2 and 3 are general views of the whole goldfish skeleton with the fifth ceratobranchial, bearing the pharyngeal teeth, painting in red. 1: Lateral view; 2: Ventral view; 3: Posterior view. 4 is the dental plate for this species with three conventional views: A: Occlusal view; B: ventral view; C: dorsal view. The dental formula for the goldfish is 4/4 (there is, on each side, one row of four teeth). Moreover, on one side, replacement teeth are visible as they are not attached to the pharyngeal bone (pointed by red arrows). The tooth shape is “compressed”.

**Figure 2:**
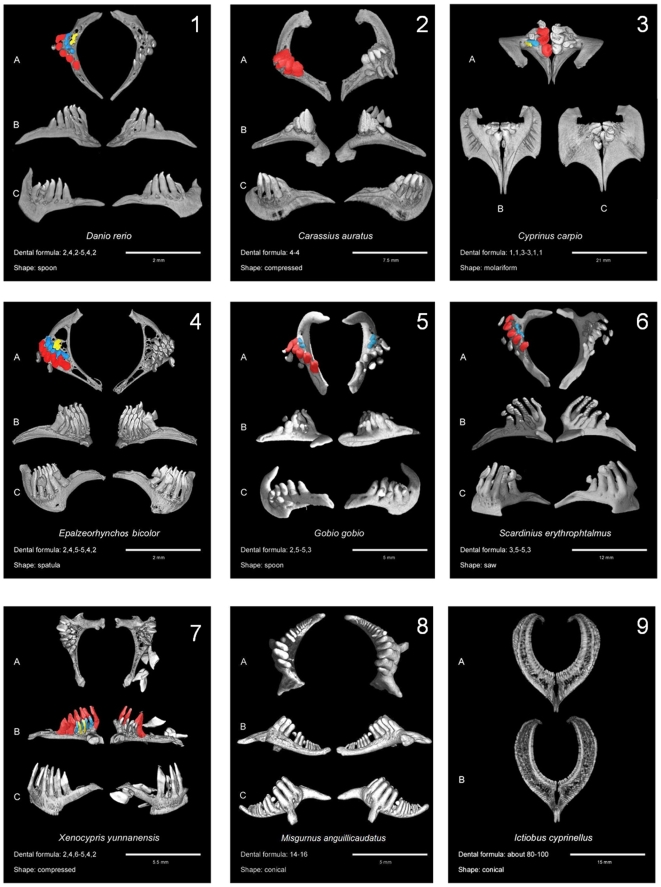
Examples of Cypriniformes dentitions obtained by microtomography and analysis of 3D reconstructions. In each case, the dental formula and the tooth shape are indicated. 1: *Danio rerio* (Rasborinae); 2,4,5; spoon. 2: *Carassius auratus* (Cyprininae); 4; compressed. 3: *Cyprinus carpio* (Cyprininae); 1,1,3; molariform. 4: *Epalzeorhynchos bicolor* (Cyprininae); 2,4,5; spatula. 5: *Gobio gobio* (Gobioninae); 2,5(−5,3); spoon. 6: *Scardinius erythrophtalmus* (Leuciscinae); 3,5; saw. 7: *Xenocypris yunnanensis* (Cultrinae); 2,3,5(−6,3,2); compressed. 8: *Misgurnus anguillicaudatus* (Cobitidae); 14–16; conical. 9: *Ictiobus cyprinellus* (Catostomidae); 55; conical. A: occlusal view; B: ventral view; C: dorsal view. Except for 8 and 9. A: anterior view; B: posterior view. For Cyprinoidea plates, teeth are painted according to the following: (i) teeth on the left side are all painted according to their row: red for ventral row, blue for mediodorsal row and yellow for dorsal row; (ii) teeth on the right side are painted only if their row displays a tooth number different from the left side (so that species displaying asymmetry are easily visible because of colours on the right half-bone).

Figure 1 shows the example of a plate for a Cyprinidae species, *Carassius auratus*, with the orientation of the dentition within the animal body. All the plates for all the species are presented in Figure S1 and a selection of diverse morphologies is presented in Figure 2. From this dataset we extracted the dental formula and we qualitatively described the tooth shape with reference to various previously described morphotypes [4], [33]. In Cyprinoidea with several tooth rows, the tooth shape was determined for teeth on the ventral row as they are the biggest and the most differentiated. By rotating 3D reconstructions, it is easy to distinguish functional teeth from replacement teeth because the latter are not bound to the bone (see teeth pointed by red arrows on Figure 1).

### Dentition patterns and tooth shape of Cypriniformes

Although the sample is small compared with the vastness of the Cypriniformes, we carefully selected candidate species in order to provide a representative view of the evolutionary diversification within the whole group.

Studied dentitions show that two organization plans can be observed within Cypriniformes, corresponding to the basal splitting into the superfamilies Cobitoidea and Cyprinoidea. Three of the four studied specimens of Cobitoidea display a single dental row with numerous conical teeth (see Figure 2, panels 8 and 9). Nevertheless, the dentition of Catostomidae differs clearly from that of Cobitidae. In Catostomidae such as *Ictiobus cyprinellus*, the teeth are arranged in a sort of “pearl necklace” comprising 55 teeth on each side whereas Cobitidae such as *Misgurnus anguillicaudatus* display fewer teeth –15– organized in a system similar to the ventral row of Cyprinoidea. Moreover, comparisons between our data and published analyses suggest that Cobitidae and Balitoridae share the same organization of dentition [8], [17]. The only known exception to this organization among Cobitoidea is *Gyrinocheilus*, the unique genus in the Gyrinocheilidae, which is toothless [8].

In contrast to the above examined families, Cyprinoidea species (Figure 2, panels 1 to 7) display a great dental diversity in terms of (i) number of tooth rows (from one to three rows), (ii) number of teeth in each row and (iii) individual tooth shapes. Concerning tooth number, a maximum is reached in the dentition of *Xenocypris yunnanensis* (Figure 2, panel 7) that encompasses up to 6 teeth on its ventral row whereas the minimum is found in *Carassius auratus* with one row of four teeth (Figure 2, panel 2). The 3D qualitative investigations of individual tooth shapes revealed the occurrence of 5 main morphotypes within the Cyprinoidea sample. These spoon, compressed, spatula, molariform and saw dental morphotypes are consistent with those defined in previous studies [4], [33]. The definitions for each tooth shape are from Chu [33]: (i) Compressed teeth are very broad teeth, with margins either straight or convex anteriorly and concave posteriorly; (ii) Spatulated teeth are teeth which are compressed but with the apical regions swollen and closely aggregated and fitted together, and with the grinding surfaces truncate, forming together a common roundish chewing area; (iii) Spoon-shaped teeth are conical teeth with a concave surface, a pointed tip and a hook; (iv) Conical teeth are simple teeth with a rounded tip; (v) Saw-shaped teeth are compressed teeth with a grinding surface as a saw, with many protuberances; (vi) Molariform teeth are crushing teeth resembling “the teeth of elephants” [33].

**Figure 3:**
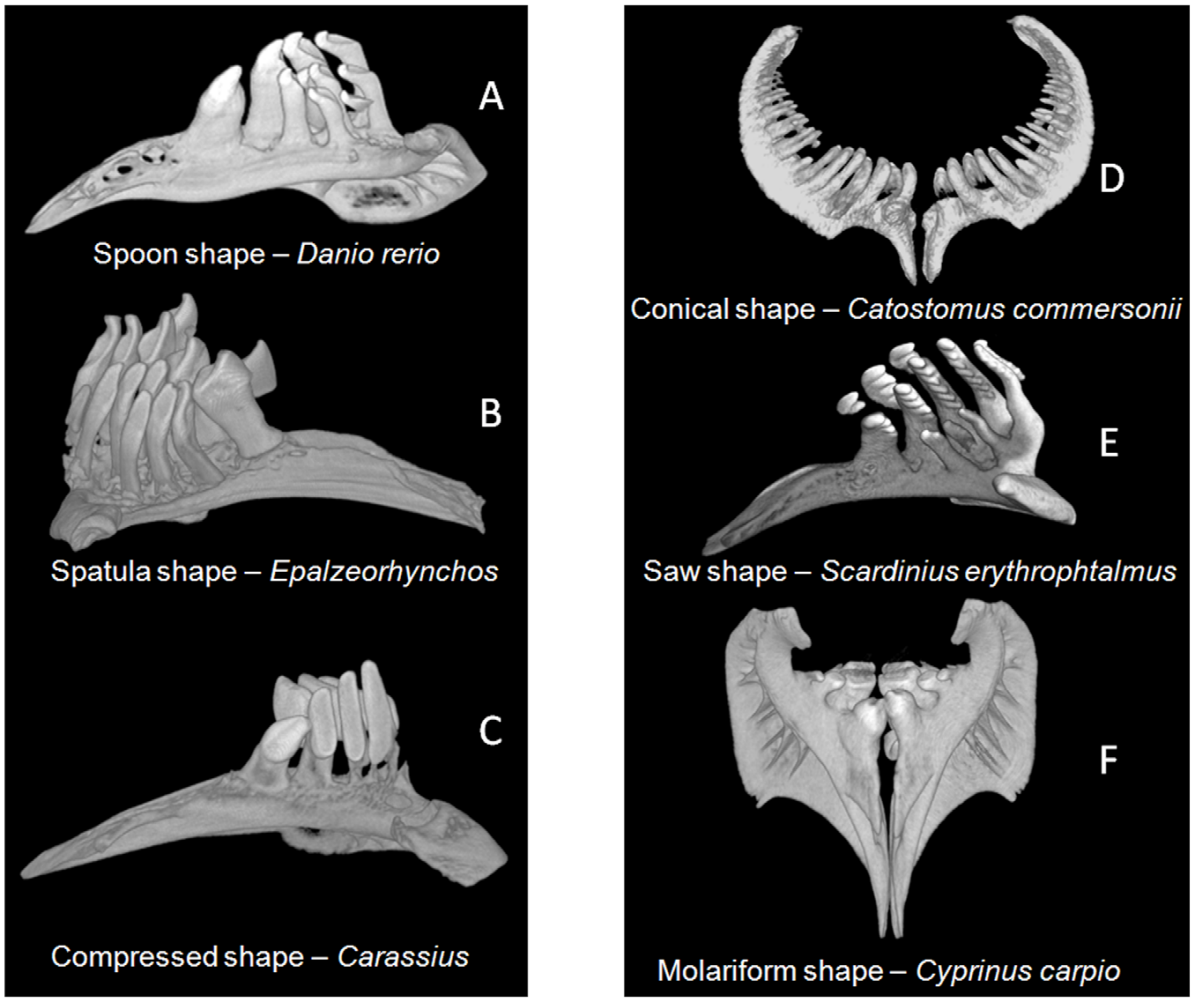
Various morphotypes of Cypriniformes teeth illustrated with examples from our dataset. A: spoon tooth shape of *Puntius semifasciolatus* (Cyprininae) with an incurved depressed region and a hook at the end of each tooth; B: spatula tooth shape of *Epalzeorhynchos bicolor* (Cyprininae), an intermediary form between the spoon and compressed morphologies, with teeth forming a grinding surface; C: compressed tooth shape of *Carassius carassius* (Cyprininae), with an elongated depressed region; D: conical tooth shape of *Catostomus commersonii* (Catostomidae); E: saw tooth shape of *Scardinius erythrophtalmus* (Leuciscinae); F: molariform tooth shape of *Cyprinus carpio* (Cyprininae).

These various morphotypes are documented in Figure 3 using examples taken from our dataset, including the conical morphotype which characterizes Cobitoidea but is not present among Cyprinoidea (Figure 3D). For instance, individual teeth are spoon-shaped in the zebrafish whereas they are molariform in the carp and compressed in the goldfish. Even if saw- or molar-shaped teeth clearly differ from one another (Figure 3E–F), spoon-, compressed- and spatula-shaped teeth appear to be more similar in shape, the spatula morphotype displaying an intermediary morphology between spoon and compressed morphotypes (Figure 3A–C).

Asymmetry is a frequent feature of Cyprinoidea pharyngeal dentition [4]. Asymmetric dentition has been documented for *Gobio gobio*, which has respectively two and three teeth on its left and right dorsal rows (Figure 2 panel 5). A similar situation is found in *Xenocypris yunnanensis*, which displays a ventral dental row with 6 teeth on the left side and 5 teeth on the right side (Figure 2 panel 7). Overall, 14 of the 42 studied species of Cyprinoidea with a determined tooth row number exhibit asymmetrical features, which is one third. This is undoubtedly not linked to replacement transient asymmetries (as a missing tooth on the ceratobranchial can be matched by a gap in the bone–see the example of *Xenocypris yunnanensis* on Figure 2 panel 7). Asymmetry is especially frequent within Leuciscinae and Rasborinae. This raises the question of the developmental mechanisms of tooth row organization that favour asymmetry inside these families. Nevertheless, asymmetric patterns can emerge within species located in clades in which most of the species exhibit a symmetrical pattern (e.g. *Puntius semifasciolatus* in Cyprininae).

**Figure 4:**
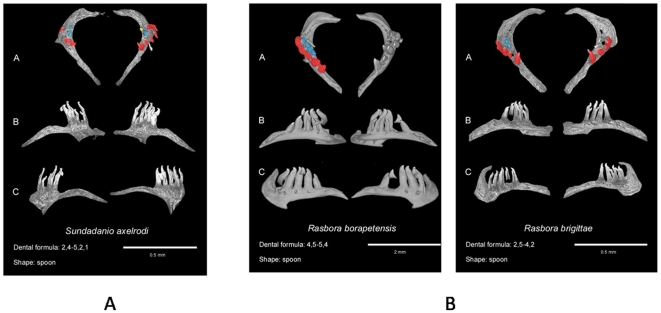
Pharyngeal dentition of minute Rasborinae. A: *Sundadanio axelrodi*, a minute Rasborinae with three tooth rows. B: *Rasbora borapetensis* and *Boraras brigittae*, two minute Rasborinae, with two tooth rows.

Cypriniformes also frequently display miniaturization, especially in Rasborinae, a subfamily that encompasses *Paedocypris progenetica*, the smallest known Vertebrate, [27], [42], [43]. We investigated the dentition in the minute Rasborinae genus *Sundadanio* and we observed that it has three tooth rows (Figure 4A), as in many normal size Rasborinae. In contrast, within the genus *Rasbora* and close species, which usually display three tooth rows, we detected only two dental rows in *R. borapetensis* and *Boraras brigittae*, considered minute species (Figure 4B).

### Analysis of Cyprinoidea tooth patterns in a phylogenetic context

**Figure 5:**
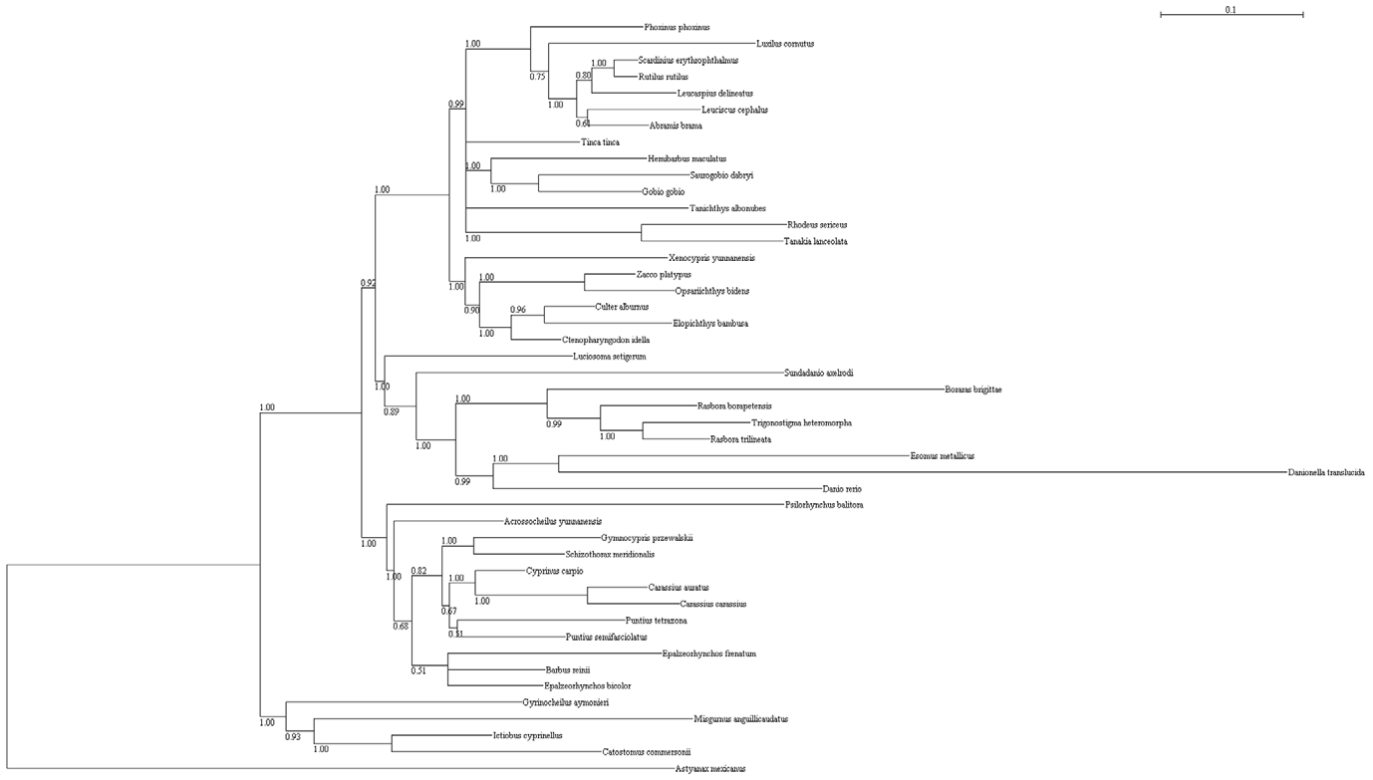
Phylogenetic relationships within Cypriniformes. The 50% majority-rule consensus tree obtained by phylogenetic Bayesian analyses, with model GTR+I+Γ, after 5,000,000 generations. Analyses were carried out with four markers whose sequences were downloaded from Genbank: cytb, rag1, rag2 and rho. Posterior probabilities are all indicated on branches. Constraints have been set so that all species are assigned to their respective monophyletic clades determined in previous studies (see Materials and Methods).

**Figure 6:**
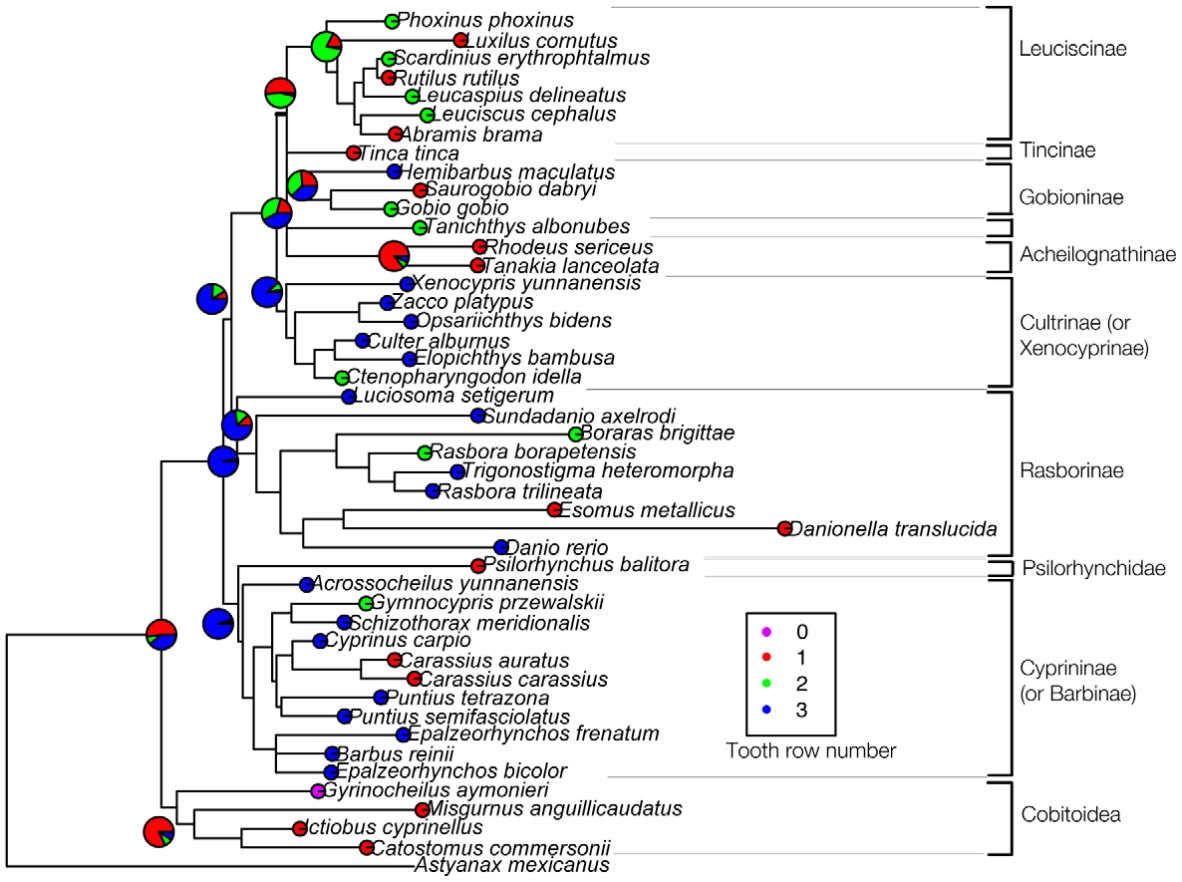
Evolution of the character “number of tooth rows” by performing ML analyses on our phylogenetic tree using BayesTraits. The variants of this character are coded with different colours: each circle in front of species' names is coloured according to the number of tooth rows either determined in our study or found in literature. The ancestral characters inferred by the ML method at different nodes of the tree are given by coloured pies. The proportion of each colour in pies represents the proportion of likelihood for each character state. For tooth row number, the state “0” was not taken into account for the ML analysis (see Materials and Methods).

**Figure 7:**
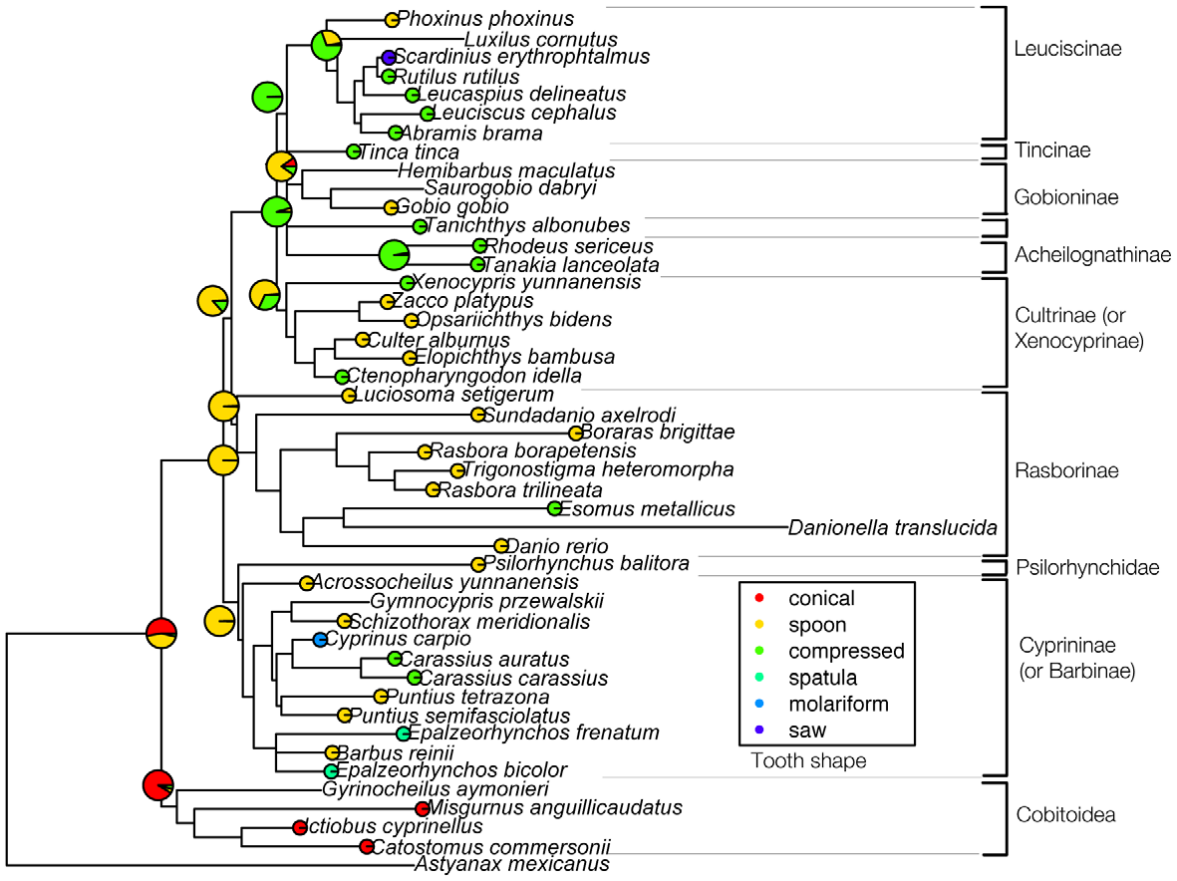
Evolution of the character “tooth shape” by performing ML analyses on our phylogenetic tree using BayesTraits. The variants of this character are coded with different colours: each circle in front of species' names is coloured according to the tooth shape determined in our study or found in literature. The ancestral characters inferred by this method at different nodes of the tree are given by coloured pies. The proportion of each colour in pies represents the proportion of likelihood for each character state. For tooth shape, the states “spatula”, “molariform” and “saw” morphotypes were not taken into account for the ML analysis (see Materials&Methods).

Tooth row number and tooth shape were analyzed by Maximum Likelihood method on our phylogenetic tree (Figure 5) with the aim of deducing ancestral conditions of these morphological characters in Cypriniformes. For each analysis, ancestral conditions were inferred at main nodes of the tree by ML using BayesTraits software, a method now widely used in literature [27], [48]–[52]. Figures 6 and 7 show the ML analysis of these characters on a tree that we built for this purpose by using sequences available on Genbank (see Materials and methods). Given that four species scanned in this study do not have any available sequence on Genbank, our phylogenetic analysis contains only 45 species, plus the outgroup *Astyanax mexicanus*. Results obtained with parsimonious reconstruction of ancestral characters using Mesquite show similar results (data not shown).

#### Tooth row number

This character varies from zero to three tooth rows within the whole order. The ML analysis allows determining the probabilities, at a given node, for each character state to be the ancestral state. BayesTraits also calculates the values of transition factors, which represent the occurrences of transitions from one character state to another. In this study, we constrained these transition factors to be all equal, which was relevant according to statistical analyses of likelihood (see Materials and methods).

Our model (Figure 6) shows unambiguously that the two super-families have different ancestral states: Cobitoidea with one tooth row versus Cyprinoidea with three tooth rows. Among Cyprinoidea, early-diverging clades which are Cyprininae, Rasborinae and Cultrinae derive from an ancestral three-row dentition whereas late-diverging clades such as Leuciscinae derive from an ancestral condition of one or two tooth rows. This general scheme can be interpreted at first glance as a general trend of reduction in tooth row number among Cyprinoidea. However, things are not so simple. First, the sub-family Gobioninae, which is a very late-diverging clade, displays species with three tooth rows (for instance, *Hemibarbus maculatus*) which shows that a secondary gain of tooth row has occurred during recent evolutionary history of Cyprinoidea.

Second, even if families are often characterized by a most frequent dental formula, this character appears very variable within various species of the subfamily (Figure 6). For instance, a two-row dentition is found for *Ctenopharyngodon idella* among Cultrinae (which classically display 3 tooth rows) and a one-row dentition for the genus *Carassius* among Cyprininae (which most often display three tooth rows). The tooth row number thus displays a high evolutionary plasticity, meaning that the Cypriniformes' dental formula is not strictly fixed at a low taxonomic level.

#### Tooth shape

The diversity of tooth shape in Cypriniformes has been categorized into 6 morphotypes (Figure 3). Among these morphotypes, conical, spoon and compressed teeth have been most frequently observed: that is why ancestral states for tooth shape have been determined with ML analysis for these three character states. The other morphotypes being very rare, we can consider that they are secondary occurrences (see Materials and methods).

Our model (Figure 7) shows unambiguously that the spoon morphotype is the ancestral state for Cyprinoidea and for early-diverging clades such as Cyprininae, Rasborinae and Cultrinae. For late-diverging clades such as Leuciscinae, the compressed morphotype is the ancestral tooth shape. Yet, as for tooth row number, this scheme is far too general. The compressed morphotype can be found in early-diverging clades (*Carassius* among Cyprininae and *Esomus* among Rasborinae) and the spoon morphotype in late-diverging clades (*Gobio* among Gobioninae). This implies that tooth shape has been frequently and convergently remodelled over the course of evolution. As for tooth row number, divergences of tooth morphotypes occur within the various families. This is the case of the dental morphotype in *Cyprinus carpio*, which is molariform, whereas the most frequently reported morphotype of Cyprininae is spoon-shaped. In the same way, *Scardinius erythrophtalmus* is the only observed Leuciscinae with saw-shaped teeth whereas most Leuciscinae display compressed teeth. It is also interesting to note that the genera *Esomus* and *Carassius* have evolved the same way with regard to both tooth row number and tooth shape. *Esomus* and *Carassius* both display a one-row dentition with compressed teeth and these genera belong to two different families in which the predominating dentition is made up of three rows of spoon-shaped teeth. This may suggest a functional coupling of these two characters in these genera.

In conclusion, more generally within Cyprinoidea, early-diverging clades are mostly species with three rows of spoon-shaped teeth whereas late-diverging clades are mostly species with one or two row(s) of compressed teeth.

## Discussion

This preliminary morphological survey of the pharyngeal dentition in 49 species selected as representative of the major clades of Cypriniformes allows us to draw several conclusions: (*i*) microtomography is an adequate tool to perform non-invasive studies of the pharyngeal dentition in Cypriniformes; (*ii*) from a primitive pattern, various strategies were adopted within the two super-families: Cobitoidea display a single row with a high number of conical teeth while Cyprinoidea display several tooth rows with few teeth in each row and a high diversity in shape; (*iii*) the fact that tooth number and tooth shape have been frequently remodeled during evolution reveals a strong plasticity of the pharyngeal dentition in Cypriniformes.

### 3D microtomography is a powerful tool to characterize pharyngeal tooth morphology

This study reveals the power of 3D microtomography in order to perform non invasive studies of Cypriniformes pharyngeal teeth. These teeth are located inside the body, therefore analysing them is destructive for the specimen. This is a main issue for museum collections as it is necessary to save those collections and find out techniques which allow studies without any damage, especially for rare specimens.

Moreover, tooth shape cannot be accurately characterized by simple observation of pharyngeal teeth (see panels of dissected specimens in this study, in Figure S1), especially for small specimens. Thus, in this latter case, tooth extraction must be followed by scanning electron microscopy which is a demanding technique. On the contrary, microtomography, as long as resolution is sufficient, lets determine tooth morphology by a simple observation of unprepared specimens.

Another positive aspect of microtomography will be the possibility to carry out a highly extensive sampling, thanks to the little time it takes (about five hours for one specimen from the scan to the virtual extraction of the bones).

### The ancestral dental formula of Cypriniformes and Cyprinoidea

Our results show that the ancestral condition of Cypriniformes cannot be determined with our limited sample of Cypriniformes species. This absence of conclusion is due to the fact that tooth row number and tooth shape are very different between the two super-families. Considering the ancestral dental formula of Cyprinoidea, the common view of a three-row dentition [33] is supported by this analysis.

Our analysis of the basal condition in Cypriniformes can be confronted to paleontological data. The oldest known fossil Cypriniformes were unearthed from the Early Palaeocene deposits of Northern America dated at about 60 Ma [53]. In Asia and Europe, the oldest Cypriniformes date from the Early Eocene at about 50 Ma [54]–[57]. The earliest North-American fossils were unambiguously identified as Catostomidae whereas the earliest Asian fossils include Catostomidae and Cyprinoidea. [54]–[57]. A frequent problem with fossil vertebrates is that teeth are the best-preserved feature during the fossilization process as dental tissues are the hardest part of the body. As a consequence, fossil Cypriniformes are frequently only represented by isolated pharyngeal teeth or fragments of pharyngeal bones and the identification of the various taxa is based quite exclusively on dental characters [58]. No other characters, independent of the dentition, can be used to assign fossil specimens to the various groups of Cyprinoidea. Thus, fossils cannot be characterized as primitive or derived forms. In certain sites where the taphonomic conditions allowed for the fossilization of the whole skeleton, complete fossils display many characters independent of the dentition. This is the case for the nearly complete skeleton of the earliest known Cyprinoidea found in China that has been assigned to the Gobioninae [56]. This suggests that some families of Cyprinoidea were already individualized at that time, which should encourage paleontologists to search for older Cypriniformes fossils.

Taken together, paleontological data indicate that, up to now, the earliest known Cypriniformes is a Catostomidae, which had a unique tooth row. But it is clear that the fossil record concerning the early evolution of Cypriniformes has to be highly improved so that a more thorough comparison of extant and fossil forms can be carried out. Notwithstanding, available data on odontogenesis of *Danio rerio* also provides interesting information. The five teeth of the ventral dental row appear completely prior to the development of the two other rows [12]. This observation indicates that the occurrence of the two other rows is a secondary process over the course of odontogenesis. All this favours the hypothesis of an ancestral pharyngeal dentition of Cypriniformes comprising a single dental row. According to our analysis, this hypothesis is as plausible as the one based on three dental rows. Thus, following that hypothesis, supplementary rows of pharyngeal teeth would have appeared quickly during the early evolution of Cyprinoidea. One argument that could support this hypothesis would be the existence of extinct or extant very-early diverging Cyprinoidea with one tooth row. According to recent phylogenetic analyses, the species *Psilorhynchus balitora* could be such a candidate but phylogenetic relationships of this species are still very unclear [26], [29]. In our study, this species is determined to be at the basis of Cyprinidae, thus displaying a basal location in the Cyprinoidea tree.

### Is there a simplification of the dentition pattern during evolution of Cyprinoidea?

Previous studies have suggested that a reduction of tooth number occurred during evolution of Cyprinoidea and that this trend was the one governing all the Cyprinoidea tooth row number evolution [4], [33]. Our ML analysis shows that there is indeed a general scheme of tooth row reduction from early-diverging clades with three tooth rows to late-diverging clades with one or two tooth row(s). Yet, as stated in the Results section, this general trend does not account for all the evolution of tooth row number in Cyprinoidea. Results showed here from morphological data clearly demonstrate that there have been multiple gains and losses of tooth rows over the course of evolution of Cyprinoidea. We found several clades close to Leuciscinae, such as Gobioninae, which can display three tooth rows. On the contrary, there are basal Cyprinoidea that display less than three tooth rows, such as *Esomus* or *Carassius*, with one tooth row.

The reduction hypothesis was also based on the general view that loss is more likely to happen during evolution than gain, which is true when several gains of complex homologous structures are considered [59]. In the case of Cypriniformes dentition, homology has never been unambiguously proved between the teeth of the different rows and among different species. This means that several appearances of two or three rows could be an example of evolutionary convergence of analogous structures, which is a frequent phenomenon during evolution [60]. Indeed, if distinct genetic modules control the development of the individual tooth and the patterning of the dental row, as it is the case in Mammals [61], [62], one can imagine independent gains and losses of tooth rows without major changes in the structure or shape of individual teeth.

Further to this, the occurrence of specimens with four tooth rows does not argue in favour of the reduction hypothesis. Several specimens have been found with four tooth rows, but they have been considered as abnormal specimens because all the other specimens of the species displayed three tooth rows [41]. The numerous reported cases of a four-row dentition occurring in Cyprinoidea demonstrate that adding a supplementary tooth row is neither a complex nor a rare event. This fits perfectly with the notion that loss is not necessarily favoured above gain, and that both processes can effectively take place. Nevertheless, previous studies favouring the reduction hypothesis considered that this four-row mutation was an atavism and thus concluded that the ancestral character could be a four-row dentition [41]. Our data reject this latter hypothesis because no species displays four tooth rows under normal development. Thus it's unlikely that no other species would have retained the four-row dentition if it had been the ancestral condition. Moreover, and it is a crucial point, the fossil record for Cyprinoidea does not indicate any species with more than three tooth rows. Therefore we state that the existence of some cases of Cyprinoidea with four tooth rows demonstrates that a supplementary dental row could easily emerge during evolution and that the atavistic interpretation is invalid.

### Functional importance of the differences in tooth number and shape

Finally, with the aim of better understanding the processes that generate the wide diversity of tooth patterns observed here, we compared our data to known Cypriniformes life history traits such as size parameters of the fishes and their feeding habits. As mentioned above, Cypriniformes include the smallest known vertebrate, the South-East Asian species *Paedocypris progenetica*, in which the adult measures less than one centimetre [27], [42]–[43]. Moreover, several other Rasborinae are minute fishes and there were several independent evolutionary trends towards miniaturization within this group [27]. Cypriniformes thus provide an excellent opportunity to evaluate if there is a correlation between the size of the fish and its dental row number. According to this hypothesis, tiny species should exhibit a reduction of their dentition. Our observations nevertheless do not support this hypothesis because we found no simple correlation between the size of the adult fish and the number of tooth rows. For instance, *Microrasbora* and *Sundadanio* that are two genera of minute Rasborinae, display three tooth rows (Figure 4A and [34]). In contrast, *Rasbora* and close species usually display three tooth rows and two minute species from this group (*R. borapetensis* and *Boraras brigittae*) only display two tooth rows (Figure 4B). Thus, no clear correlation exists between the number of tooth rows and the size of the fish. More generally, it is difficult to conclude on the existence of a link between dental row number and size parameters such as standard length. Indeed, Rasborinae species often display three tooth rows and they are among the smallest Cyprinoidea (less than ten centimetres) whereas Leuciscinae species commonly display one or two tooth row(s) and they are generally big fishes (more than thirty centimetres). It would nevertheless be interesting to more extensively document Cypriniformes size parameters and compare tooth organization with more relevant parameters such as head length or ceratobranchial length, as the standard length is probably not the most appropriate parameter to be considered.

Concerning feeding habits, few precise data are available for Cypriniformes [63]. In fact, the only useful data for such a comparison should come from fishes taken from their natural habitat since fishes kept in fisheries or in ponds do not necessarily have the same behaviour as their natural congeners. Most available data from wild specimens come from simple observations of species from common European rivers that are mainly considered as omnivorous [35]. Highly specialized tooth shapes, such as the saw morphotype in *Scardinius eryhtrophtalmus*, obviously suggests an adaptation to a special diet but no precise data allow us to substantiate this assumption. There are also close genera with divergent tooth shapes, such as *Carassius* with compressed teeth and *Cyprinus* with molariform teeth, which suggests that feeding habits could be a highly diversifying evolutionary factor. Once again, available data on wild specimens are scarce. It would thus be of great interest to document more precisely the diet of such species by analyzing stomach contents of wild specimens.

### Perspectives

With respect to the techniques used, investigation of dentition using microtomography was by far more revealing than microscopic examination of dissected or cleared specimens. Furthermore, X-ray image acquisitions are not invasive, a fundamental consideration for material borrowed from museum collections. Laboratory microtomographs proved to be able to image in 3D the tooth number and tooth shape in small to large size Cypriniformes (Figure 2 and Figure S1). For high quality scan, minute specimens or larvae, it is preferable to use synchrotron sources. Microtomography also makes it easier to image and distinguish functional teeth from replacement teeth because replacement teeth are not attached to the pharyngeal bones and these teeth could be lost or omitted in case of dissection.

The present study clearly shows that a systematic survey of pharyngeal dentition patterns in Cypriniformes using 3D microtomography is feasible and will allow for a better evaluation of the factors that could have played a role in the stunning diversity observed in these fishes. Indeed, our ML analyses of Cypriniformes' ancestral dental characters is based on a small data set with regards to the size of the order Cypriniformes and certainly requires a more complete analysis from a wider sampling. We believe that the Cypriniformes' pharyngeal dentition offers a very good model to study the factors implicated in the evolutionary diversification of complex morphological structures. Indeed, Cypriniformes include both a very strong diversity of patterns coupled with the existence of an excellent functional model for developmental biology and genomics, the zebrafish *Danio rerio*.

Cypriniformes tooth morphology will certainly benefit from a better understanding of the genetic processes underlying the diversity of pharyngeal teeth among Cypriniformes. For this, it will be useful to study developmental mechanisms underlying tooth development of other Cyprinoidea for comparisons with *Danio rerio*, especially species with a different number of tooth rows. It has already been shown in mouse mutants that it is possible to determine genes involved in the number of tooth rows [64]. It would also be interesting to produce mutants of *Danio rerio* with abnormal numbers of tooth rows for which the mutation could be characterized, in order to determine a set of genes that could be important for tooth row organization.

Another interesting prospect of this work would be to perform a quantitative analysis of tooth shape based on 3D data, instead of the relatively simple qualitative description that we have performed in this paper. Such an approach has already been carried out with *Danio rerio* [65] in order to test differences in tooth size and shape during replacement tooth cycles. Quantitative analyses of tooth size and shape have also been carried out to infer evolutionary trends, for instance with rodents [66]. This would allow for better understanding of the various directions in the morphospace in which the changes occurred, the constraints that are shaping these variations and to better understand the processes of evolutionary convergence of tooth shape [67]. This could contribute to bridge a gap between developmental studies and classical morphological analysis and to establish Cypriniformes as a useful integrated model for dentition Evo/Devo studies.

## Materials and Methods

### Cypriniformes sampling

The Cypriniformes specimens analyzed here belong to 49 different species: (i) 31 species have been scanned especially for the present study; (ii) 3 species have been dissected and dentition has been imaged by traditional microscopy; (iii) the dentition of the 15 other species has been taken from the literature. All Cypriniformes analyzed in this study are listed in Table 1. We chose specimens belonging to the two large superfamilies: Cobitoidea and Cyprinoidea. For Cobitoidea we sampled 3 of the 4 families: Catostomidae, Cobitidae, Gyrinocheilidae. Given the current questions regarding the evolution of dentition in Cyprinidae, we focused our analysis on this super-family, and sampled specimens from each of its sub-families: Rasborinae, Cyprininae, Cultrinae, Acheilognathinae, Gobioninae, Leuciscinae and Tincinae. The sampling was also focused on minute Rasborinae. Several Cypriniformes came from different pet shops as they comprise many species that are very common among exotic fish breeders. As indicated in Table 1, many Cypriniformes came from the National Museum of Natural History of Paris (MNHN), the Natural History Museum of London (NHM) as well as from the Laboratory of Animal Sciences of Nancy (LSA). Reference numbers are indicated in Table 1 for specimens loaned from museums of Paris and London.

**Table 1:** Cypriniformes species included in this study.

Scientific name	Clade	Dental formula	Tooth shape	Origin of specimen^1^
***Psilorhynchus balitora***	Psilorhynchidae	4	Spoon	MNHN, 1931-056
***Boraras brigittae***	Rasborinae	2,5-(4,2)	Spoon	Pet shop
***Danio rerio***	Rasborinae	2,4,5	Spoon	Pet shop
*Danionella translucida*	Rasborinae	1 row		[38]
***Esomus metallicus***	Rasborinae	5-(4)	Compressed	MNHN, 1987-1355
***Laubuca laubuca***	Rasborinae	2,4,5-(4,4,2)	Spoon	MNHN, 1983-0214
***Luciosoma setigerum***	Rasborinae	2,4,5	Spoon	MNHN, 1994-0232
***Opsaridium christyi***	Rasborinae	3,5	Spoon	MNHN, 1986-0416
***Rasbora borapetensis***	Rasborinae	4,5	Spoon	MNHN, 1983-0249
***Rasbora einthoveni***	Rasborinae	2,4,5	Spoon	MNHN, 1986-0228
***Rasbora trilineata***	Rasborinae	2,4,5	Spoon	Pet shop
***Sundadanio axelrodi***	Rasborinae	2,4-(5,2,1)	Spoon	MNHN, 1982-0681
***Trigonostigma heteromorpha***	Rasborinae	2,5-(5,2,2)	Spoon	Pet shop
***Acrossocheilus yunnanensis***	Cyprininae	2,3,5	Spoon	MNHN, 1949-0037
***Barbus reinii***	Cyprininae	2,3,5	Spoon	[34]
***Carassius auratus***	Cyprininae	4	Compressed	Pet shop
***Carassius carassius***	Cyprininae	4	Compressed	LSA
***Cyprinus carpio***	Cyprininae	1,1,3	Molariform	LSA
***Gymnocypris przewalskii***	Cyprininae	2 rows		[34]
***Epalzeorhynchos bicolor***	Cyprininae	2,4,5	Spatula	Pet shop
***Epalzeorhynchos frenatum***	Cyprininae	2,4,5	Spatula	Pet shop
***Puntius semifasciolatus***	Cyprininae	2,3,4-(5,3,2)	Spoon	Pet shop
***Puntius tetrazona***	Cyprininae	2,3,4	Spoon	Pet shop
***Schizothorax meridionalis***	Cyprininae	2,3,5	Spoon	[34]
***Ctenopharyngodon idella***	Cultrinae	2,4	Compressed	[4]
***Culter alburnus***	Cultrinae	2,4,4-(5,4,2)	Spoon	MNHN, 1934-0213
***Elopichthys bambusa***	Cultrinae	2,4,5	Spoon	[33]
***Opsariicthys bidens***	Cultrinae	1,4,5	Spoon	[33]
***Xenocypris yunnanensis***	Cultrinae	2,3,5-(6,3,2)	Compressed	MNHN, 1949-0042
***Zacco platypus***	Cultrinae	1,4,5	Spoon	[33]
***Rhodeus sericeus***	Acheilognathinae	5	Compressed	Pet shop
***Tanakia lanceolata***	Acheilognathinae	5	Compressed	MNHN, 1984-0419
***Tanichthys albonubes***		1,4-(5,1)	Compressed	Pet shop
***Gobio gobio***	Gobioninae	3,5-(5,2)	Spoon	LSA
***Hemibarbus maculatus***	Gobioninae	1,3,5		[33]
***Saurogobio dabryi***	Gobioninae	5		[33]
***Tinca tinca***	Tincinae	4-(5)	Compressed	LSA
***Abramis brama***	Leuciscinae	5	Compressed	[37]
***Leucaspius delineatus***	Leuciscinae	1,4-(5,1)	Compressed	LSA
***Leuciscus cephalus***	Leuciscinae	2,5	Compressed	[35]
***Luxilus cornutus***	Leuciscinae	4		[35]
***Phoxinus phoxinus***	Leuciscinae	2,4-(5,2)	Spoon	[39]
***Rutilus rutilus***	Leuciscinae	5-(6)	Compressed	LSA
***Scardinius erythrophtalmus***	Leuciscinae	3,5	Saw	LSA
***Carpiodes microstomus***	Catostomidae	50	Conical	MNHN, 1905-0340
***Catostomus commersonii***	Catostomidae	30	Conical	MNHN, 1994-0301
***Ictiobus cyprinellus***	Catostomidae	55	Conical	MNHN, 2002-0352
***Misgurnus anguillicaudatus***	Cobitidae	14-(16)	Conical	Pet shop
*Gyrinocheilus aymonieri*	Gyrinocheilidae	0-0		[8]

Dental formulas and tooth shapes in various Cypriniformes determined in this study by microtomography and/or clearing (species in bold letters) or found in literature.

The dental formula of one half-bone is written when the species displays a symmetrical dental formula whereas in the case of asymmetrical formulas, the formula of the right half-bone is in parentheses. Dental formulas were determined as explained in the introduction, i.e. by counting the number of teeth on each row from the most ventral to the most dorsal row (and the inverse for the right bone). For data coming from the literature, tooth shape was sometimes not available. For species scanned or dissected in this study, origin of the specimen is written (petshop, MNHN for the National Museum of Natural History of Paris, NHM for the National History Museum of London, LSA for the Laboratory of Animal Sciences of Nancy) as well as reference numbers for museum specimens. For data coming from literature, the reference in which data were found is given.

1 The reference for loaned specimens from museums is provided or bibliographical references for species that were not scanned in this study but used in the parsimonious analysis.

### Phylogenetic analyses

As mentioned in the Results section, we built our own phylogenetic tree in this study, not with the intention to publish a new Cypriniformes phylogeny, but as a necessary step to reconstruct ancestral characters with BayesTraits [48], a method which requires branch lengths. That is why we did not carry out any sequencing effort but we used available sequences. We worked with four neutral markers: cytochrome b (cytb), recombination activating gene 1 (rag1), recombination activating gene 2 (rag2) and rhodopsin (rho). Four species that we scanned in this study were not used for the phylogenetic analysis as no sequence is available on Genbank: *Carpiodes microstomus*, *Rasbora einthoveni*, *Laubuca laubuca* and *Opsaridium christyi*. Thus, 45 species were included in our phylogenetic analysis, plus the outgroup *Astyanax mexicanus*. All sequences used for our phylogeny were downloaded from Genbank. Accession numbers are indicated in Table 2. Sequences were aligned using Seaview [68]: in order to limit the number of gaps, we kept 1142 base pairs for cytb, 1497 for rag1, 1251 for rag2 and 485 for rho. All sequences were concatenated for each species. Phylogenetic analysis was carried out with Mr Bayes [69]. The parameters were as followed: lset nst = 6, rates = invgamma. Sequences were partitioned with respect to the four genes and the three codon positions. The command “unlink” was used to set different model parameters for the different partitions. Four MCMC chains were performed with 5,000,000 generations, sampling one tree per 100 replicates. We discarded the 10,000 first sampled trees as burn-in after diagnostic of convergent likelihood. These trees were used to construct a 50% majority rule consensus tree with posterior probabilities for each node. Our phylogenetic tree was then compared to previously published ones in order to determine any incongruence. Three taxa were not assigned to their usual clades in our analysis: *Luciosoma setigerum* was determined as an outgroup of the Rasborinae, *Xenocypris yunnanensis* was determined as an outgroup of the Cultrinae and *Gyrinocheilus aymonieri* was determined as an outgroup of the order Cypriniformes. As previous papers have shown that the two first taxa are respectively members of Rasborinae and Cultrinae [70], [71] and as several papers have proved the monophyly of the majors clades within Cypriniformes [23]–[29], we decided to set constraints in our Bayesian analysis so that all species in our sample are correctly assigned to their monophyletic clade (Cobitoidea, Cyprininae, Rasborinae, Cultrinae, Gobioninae, Leuciscinae, Acheilognathinae).

**Table 2:** Genbank accession numbers for sequences used in this study.

Scientific name	Cytb	Rag1	Rag2	Rho
*Psilorhynchus balitora*		EF056369		
*Boraras brigittae*	EU241414			EU241347
*Danio rerio*	EU241427	U71093	U71094	EU241362
*Danionella translucida*	FJ753515	FJ753544		
*Esomus metallicus*	EU241441	FJ753531		EU241376
*Luciosoma setigerum*		EU292704		FJ531352
*Rasbora trilineata*	EU241470			EU241404
*Rasbora borapetensis*	EU241463			EU241398
*Sundadanio axelrodi*	EU241474	FJ753540		EU241408
*Trigonostigma heteromorpha*	EU241477	FJ753530		EU241411
*Epalzeorhynchos bicolor*	AJ388457	GQ913456		GQ913508
*Epalzeorhynchos frenatus*		EF056360	DQ366943	GQ913509
*Acrossocheilus yunnanensis*	AF051857			
*Barbus reinii*	AF145946			
*Carassius auratus*	FJ169954	EF186007	DQ366941	
*Carassius carassius*	FJ167428			
*Cyprinus carpio*	DQ868875	EF458304	DQ366994	
*Gymnocypris przewalskii*	DQ309362	EU711149	DQ366954	
*Puntius semifasciolatus*	EU241460	DQ366951		EU241394
*Puntius tetrazona*			DQ366938	
*Schizothorax meridionalis*	AY954287		DQ366989	
*Ctenopharyngodon idella*	AF420424	EF178284	DQ366996	
*Culter alburnus*			DQ367004	
*Elopichthys bambusa*	AY744501		DQ367016	
*Opsariicthys bidens*	AY245090		DQ367014	
*Xenocypris yunnanensis*	AF036208			
*Zacco platypus*	AF309085	EF452848	DQ367010	EF452917
*Rhodeus sericeus*	Y10454			
*Tanakia lanceolata*	AB208543			
*Tanichthys albonubes*	EU241475	FJ531253	DQ367023	EU241409
*Gobio gobio*	AY953007	EU292689	DQ367015	
*Hemibarbus maculatus*	AY952990			
*Saurogobio dabryi*	AY953011		DQ367020	
*Tinca tinca*	AJ555551	EU711162	DQ367029	
*Abramis brama*	AJ555548	EU711103		
*Leucaspius delineatus*	AJ388459			
*Leuciscus cephalus*	GU182336			
*Luxilus cornutus*	U66597			
*Phoxinus phoxinus*	EF094550		DQ367022	FJ197065
*Rutilus rutilus*	FJ025074	EU711126	DQ367003	
*Scardinius erythrophtalmus*	EF105295	EU409628		EU409656
*Catostomus commersoni*	AY279397	EU409612		EU409638
*Ictiobus cyprinellus*	FJ226308			
*Misgurnus anguillicaudatus*	EF088651	EU670842		
*Gyrinocheilus aymonieri*	DQ105256	EF056390		EU409663
*Astyanax mexicanus*	FJ439346	FJ439461		

Accession numbers for all molecular sequences used in our study for the building of the phylogenetic tree. All sequences were downloaded from Genbank.

### X-ray microtomography

Fishes were scanned either using a conventional microtomograph or at the European Synchrotron Radiation Facility (ESRF, Grenoble, France), which is a third generation synchrotron [46]. The conventional microtomograph was a Viscom X8050-16 high resolution (voxel size used from 5 µm to 20 µm) at the iPHEP (International Institute of Paleoprimatology, Human Evolution and Paleoenvironments) in Poitiers. Cypriniformes were scanned with the following settings: 200 µA and from 80 to 120 kV depending on the size of the individual (the bigger the fish, the higher the voltage due to the increased thickness of the element the *X-*rays must traverse). The tomographic reconstruction was performed using the software attached to the machine. Small Cypriniformes (less than 50 mm) were scanned on the beam line BM05 at the ESRF (European Synchrotron Radiation Facility) of Grenoble. We used isotropic voxel sizes of respectively 5.4 µm and 0.691 µm (depending on the size of the specimens). We used a monochromatic beam at energy of 20 keV for the largest samples (monochromatization done with a double Si111 Bragg system) and 18 keV for the smallest ones (monochromatization done with a double multilayer monochoromator). Ring artefacts and tomographic reconstructions were performed using ESRF inhouse tools.

### Morphological analyses

Reconstructed volumes were analyzed with VGStudioxMax 1.2.1 (Volume Graphics, Heidelberg, Germany) and the fifth ceratobranchial was extracted for each sample using the 3D magic wand growing region tool and manual adjustments. Ancestral states of tooth row number and tooth shape were reconstructed for different nodes of our phylogenetic tree by Maximum Likelihood method with BayesTraits [48]. BayesTraits reconstructs the ancestral characters at each node of the tree by finding the lowest likelihood value for the whole data, and gives the transition factors corresponding to this likelihood value. Those transition factors represent the values for each transition from one character state to another in the whole tree. Then, it is possible to build models in which transition factors can be constrained. For instance, transition factors can be constrained to be all equal or one transition factor can be constrained to be zero. BayesTraits gives the likelihood value associated with the model. Statistical tests can be carried out to compare likelihood values between different models, in order to know if likelihood values are statistically different or not. For this study, there was no statistical difference between the complex model (no constraint) and a simpler model, which is that all transition factors are constrained to be equal. Once a model has been chosen, it is possible to extract, for each node of the tree, the likelihood value associated with each character state at a given node. These values were plotted in our phylogenetic tree, using pies. Moreover, because of a small number of species compared to the number of character states for both tooth row number and tooth shape, rare states (which are “0” for tooth row number and “spatula”, “molariform” and “compressed” morphotypes for tooth shape) tend to be determined as ancestral states by BayesTraits, no matter which model is used, which is very unlikely as these character states are almost all restricted to only one species of our dataset. Consequently, based on the parsimonious hypothesis that these rare character states cannot be considered as ancestral, they were discarded from the ML analysis of ancestral characters with BayesTraits but they are represented for convenience on Figures 6 and 7.

### Fish clearing and staining

Cypriniformes were cleared and stained according to Taylor and van Dyke [45]. For relatively big individuals (more than 5 cm) that are long to clear entirely, they were stained with Alizarin red (which colours bones) and the fifth ceratobranchial bearing pharyngeal teeth was extracted by dissecting the samples. The 5^th^ ceratobranchial was then observed with binoculars and pictures were taken with a photograph included in the binoculars.

## Supporting Information

Figure S1 Plates of the 34 Cypriniformes scanned or dissected in this study. 31 species were surveyed by 3D microtomography. The corresponding plates present the pharyngeal dentition in three views (A: occlusal view; B: dorsal view; C: ventral view), except for Catostomidae (A: anterior view; B: posterior view) and Psilorhynchus (only occlusal view). For the four dissected specimens, one view is given for each plate (the type of view is written); the view was chosen in order to best determine the dental formula. For all plates, the name of the species and its dental formulas are written, as well as a scale giving the size of the fifth ceratobranchial arch. Tooth shape is only available for species surveyed by microtomography. In each plate, the different tooth rows are differentiated using colours (from the most ventral to the most dorsal rows: red, blue, yellow, green) on the left side. Asymmetrical patterns are shown by the use of colours on the right side, for the rows which display a different number of teeth.
(4.71 MB TIF)
