# Supplementary figures and images for: Evolutionary Trends of the Pharyngeal Dentition in Cypriniformes (Actinopterygii: Ostariophysi)
Source: PLoS One. 2010 Jun 24;5(6):e11293. doi: 10.1371/journal.pone.0011293 (PMC2892034; doi:10.1371/journal.pone.0011293)

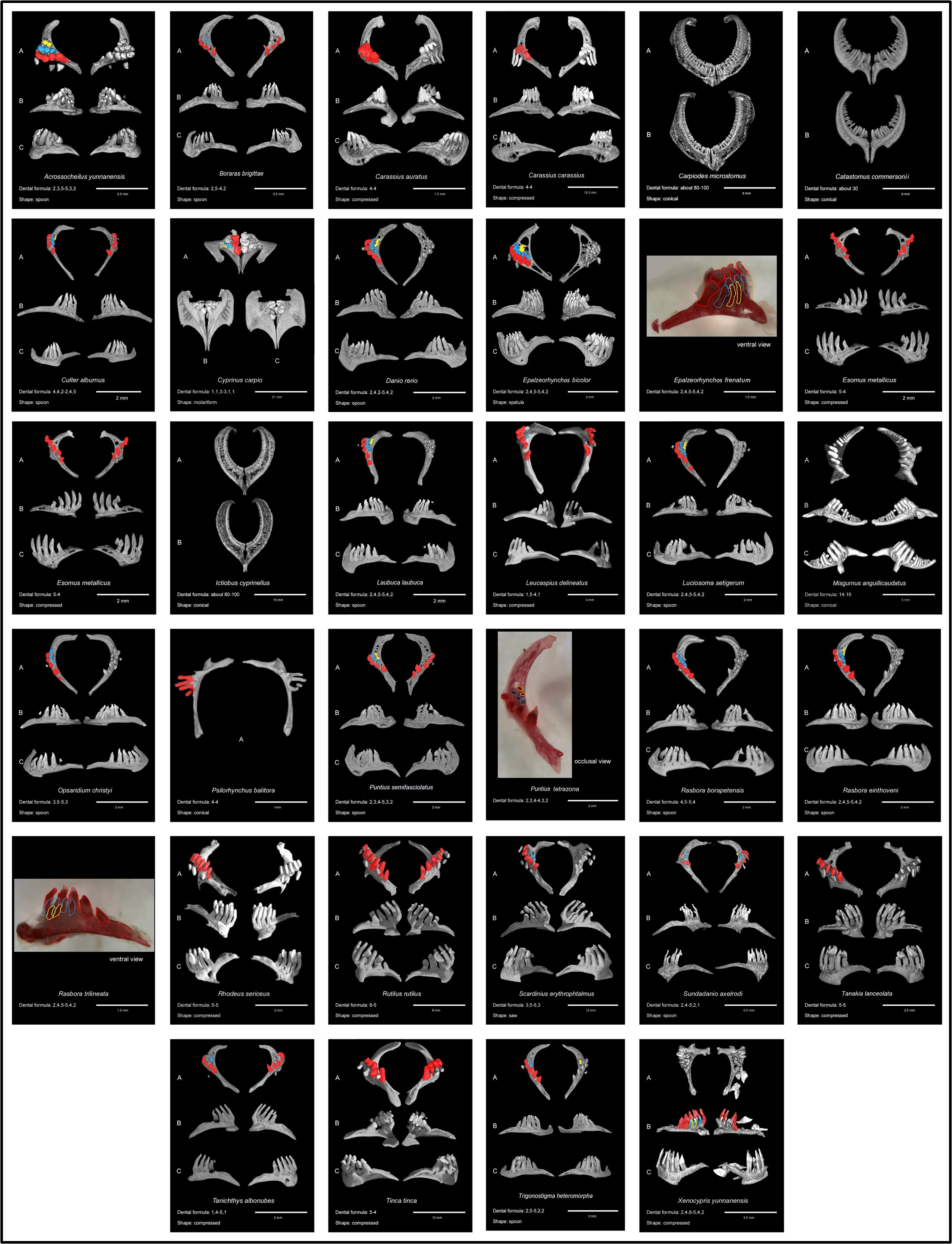

Supplement: Figure S1 — Plates of the 34 Cypriniformes scanned or dissected in this study. 31 species were surveyed by 3D microtomography. The corresponding plates present the pharyngeal dentition in three views (A: occlusal view; B: dorsal view; C: ventral view), except for Catostomidae (A: anterior view; B: posterior view) and Psilorhynchus (only occlusal view). For the four dissected specimens, one view is given for each plate (the type of view is written); the view was chosen in order to best determine the dental formula. For all plates, the name of the species and its dental formulas are written, as well as a scale giving the size of the fifth ceratobranchial arch. Tooth shape is only available for species surveyed by microtomography. In each plate, the different tooth rows are differentiated using colours (from the most ventral to the most dorsal rows: red, blue, yellow, green) on the left side. Asymmetrical patterns are shown by the use of colours on the right side, for the rows which display a different number of teeth. (4.71 MB TIF) [file pone.0011293.s001.tif]
